# Cost-effectiveness analysis of sugemalimab vs. placebo, in combination with chemotherapy, for treatment of first-line metastatic NSCLC in China

**DOI:** 10.3389/fpubh.2022.1015702

**Published:** 2022-11-03

**Authors:** Wei Li, Li Wan

**Affiliations:** Department of Pharmacy, Maternal and Child Health Hospital of Hubei Province, Tongji Medical College, Huazhong University of Science and Technology, Wuhan, China

**Keywords:** cost-effectiveness, NSCLC, sugemalimab, GEMSTONE-302 trial, first-line treatment, Markov, PD-L1

## Abstract

**Objective:**

The purpose of this study was to estimate the cost-effectiveness of sugemalimab plus chemotherapy (SC) vs. placebo plus chemotherapy (PC), as the first-line treatment for patients with non-small cell lung cancer (NSCLC) in China.

**Material and methods:**

A three-state Markov model with a cycle of 3 weeks was built to assess the incremental cost-effectiveness ratio (ICER) of SC vs. PC as first-line treatment for patients with NSCLC over a 10-year horizon from Chinese health care perspective. Time-dependency transition probability and safety data were derived from a multicenter, randomized, double-blind, phase 3 clinical trial performed in China (GEMSTONE-302). Primary model outcomes included the costs in US dollars and health outcomes in quality-adjusted life-years (QALYs) and the ICER under a willingness-to-pay (WTP) threshold of $37,663/QALYs. Deterministic, scenario and probabilistic sensitivity analysis were employed to investigate the robustness of model outcomes.

**Results:**

In base-case analysis, compared with PC, first-line SC for intention-to-treat (ITT) population gained an additional 0.57 QALYs with an incremental cost of $62,404.15, resulting in an ICER of $109,480.97/QALYs gained. When a patient assistance program (PAP) was available, the ICER decreased to $52,327.02/QALYs. In subgroup analysis, the ICER values were above the WTP threshold with or without PAP. Sensitivity analysis results suggested that the model outcomes were reliable.

**Conclusion:**

From the perspective of Chinese healthcare system, the SC was not cost-effective in comparison to PC as first-line treatment for NSCLC, regardless of PD-L1 tumor expression level and pathological subtype.

## Introduction

Lung cancer is a malignant tumor with the highest incidence and mortality in China (1), and non-small cell lung cancer (NSCLC) is the most common subtype of lung cancer, accounting for about 85% of all lung cancers (2). In recent years, the emerging immunotherapy, targeting against programmed death 1 (PD-1)/programmed death ligand 1 (PD-L1) signaling, has changed the treatment pattern of advanced NSCLC (3).

Sugemalimab (suge), a newly developed fully human monoclonal antibody against PD-L1, in combination with pemetrexed plus carboplatin for non-squamous (NSQ) NSCLC and in combination with paclitaxel plus carboplatin for squamous (SQ) NSCLC, exhibiting exciting clinical benefit and acceptable safety profile (4), was approved in China for the first-line treatment of epidermal growth factor receptor (EGFR) gene mutation and anaplastic lymphoma kinase (ALK) negative metastatic NSQ and SQ NSCLC (5). This is the first PD-L1 antibody to be approved as a first-line treatment for both advanced NSQ and SQ NSCLC.

GEMSTONE-302 trail (4, 6) revealed that, compared to placebo plus chemotherapy (PC), the sugemalimab plus chemotherapy (SC) remarkably extended the median overall survival (mOS) by 8.5 months (mOS, 25.4 vs. 16.9 months; hazard ratio (HR), 0.65; 95% CI, 0.50–0.84) and prolonged median progression-free survival (mPFS) by 4.1 months (mPFS, 9.0 vs. 4.9 months; HR, 0.48; 95% CI, 0.39–0.60) for patients with NSCLC, regardless of PD-L1 expression level and tumor pathological type. China, as the biggest developing country with an accelerating process of population aging, have to pay more attention to how to maximize the role of limited medical resources. Sugemalimab, as a newly innovative drug, the price of which is often relatively higher due to the large investment in early research and development. Although sugemalimab brought meaningful clinical benefits, but also increased the economic burden of patients' families, especially middle- and low-income families. Whether its cost is proportional to its clinical value in China was insufficiently considered.

In this study, we built a decision-analytic model and used it to compare the cost-effectiveness of SC with PC for first-line treatment of metastatic NSCLC from the perspective of China's healthcare system. We hope this will help the health policymakers, patients and physicians select the right strategy from an economic perspective.

## Materials and methods

### Model structure and outcomes

**Figure 1:**
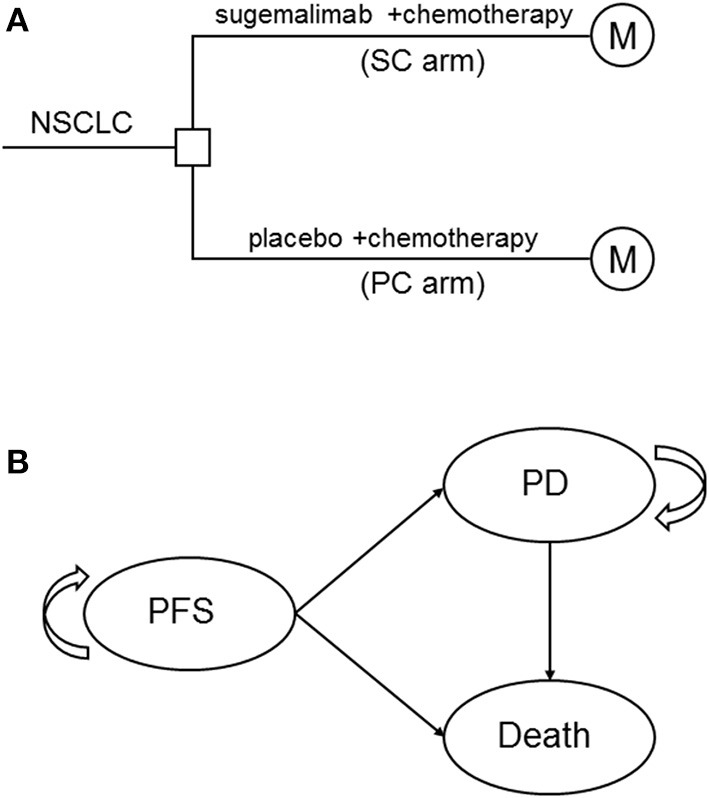
The schematics of the decision tree **(A)** and the Markov state transition model **(B)**.

A mathematical model that combined decision tree and Markov model was constructed to estimate the clinical and economic outcomes of sugemalimab vs. placebo, in combination with platinum-based chemotherapy as first-line treatments of metastatic NSCLC (Figure 1A). A three states Markov model, including three mutually exclusive disease-related health states: progression-free survival (PFS), progressed disease (PD), and death was built by using TreeAge Pro 2011 (TreeAge Software, Williamstown, MA) (Figure 1B). It was assumed that all patients entered the model in the PFS state and then either remained in the same state or transited to the other states during a Markov cycle length of 3 weeks. Patients in initial state were assumed to be 63 years old, because this reflected the median age of previously treated patients included in GEMSTONE-302. The time horizon for the model was set to 10 years, within this time frame 85 and 97% of patients died in SC arm and PC arm, respectively.

The primary model outcomes were the corresponding total costs of two therapeutic regimens, quality-adjusted life years (QALYs), and the incremental cost-effectiveness ratio (ICER). Both costs and utility values were discounted at an annual rate of 5% for base-case analysis, according to guideline for health economic evaluations in China (7). All costs were converted into 2021 US dollars (US 1$ = CNY ¥6.45). Three times of the per capita gross domestic product (GDP) of China in 2021 (US$37,663/QALYs) was used as the willingness-to-pay (WTP) threshold to assess the cost-effectiveness of the two competing strategies (7).

### Clinical data and transition probabilities

Clinical efficacy and safety data were obtained from a multicenter, randomized, double-blind, phase 3 trial done in China (GEMSTONE-302, ClinicalTrials.gov number, NCT03789604). In this trail, eligible patients were randomly assigned (2:1) to SC arm or PC arm. The ratio of SQ to NSQ NSCLC in both groups was 0.4 to 0.6 (SQ: NSQ = 0.4: 0.6). Patients with NSQ received sugemalimab (1,200 mg, Q3W) or placebo, plus carboplatin (AUC 5 mg/mL/min, Q3W) and pemetrexed (500 mg/m^2^, Q3W), for up to four cycles, followed by maintenance treatment with pemetrexed plus either sugemalimab or placebo. Patients with SQ received sugemalimab (1,200 mg, Q3W) or placebo, plus carboplatin (AUC 5 mg/mL/min, Q3W) and paclitaxel (175 mg/m^2^, Q3W), for up to four cycles, followed by maintenance treatment with sugemalimab or placebo. Sugemalimab or placebo treatment continued for up to 35 cycles, unless disease progression or unacceptable toxicity.

**Figure 2:**
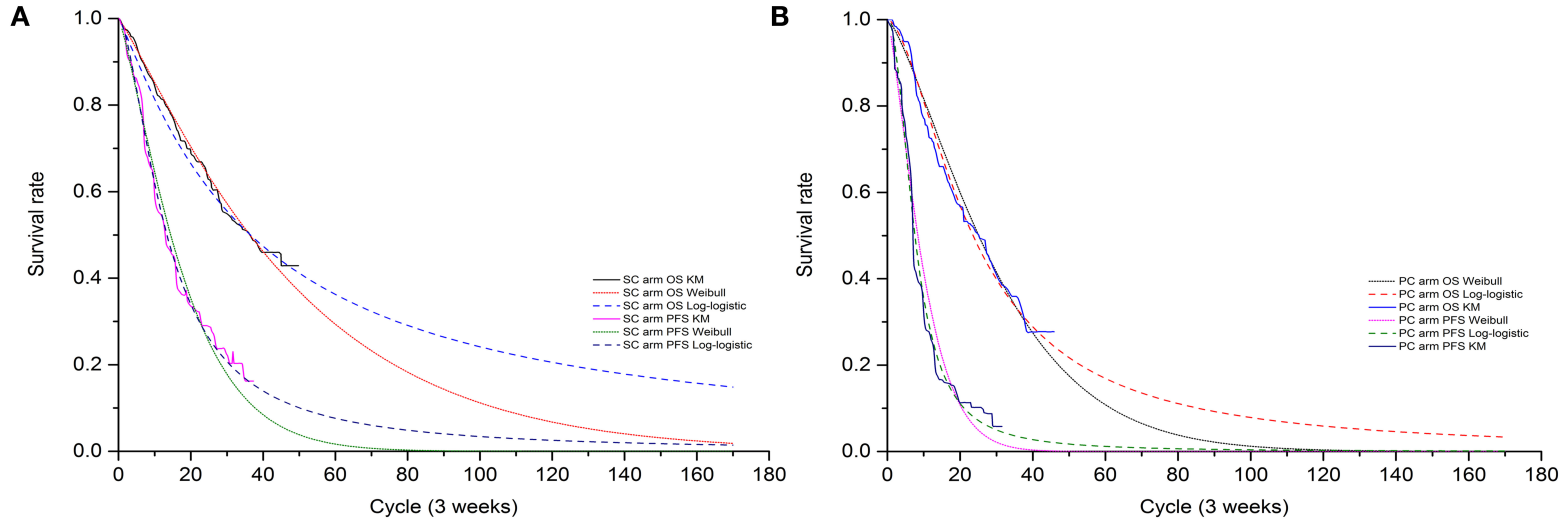
The exploration and fitting of PFS and OS curves in SC arm **(A)** and in PC arm **(B)**.

Transition probabilities were estimated from the OS and PFS Kaplan-Meier (K-M) curves of the GEMSTONE-302 trial. First, GetData Graph Digitizer software (version 2.26) was employed to extracted the graphical data from published K-M curves. Second, R software (version 4.0.3) was used to reconstruct the individual patient data (IPD) derived from previous study (8). Third, to extrapolate survival curve beyond the observation period, we considered the following two-parametric distributions to fit the IPD: Exponential, Weibull, Gompertz, Log-logistic, and Log-normal. The choice of the model to use was based on Akaike's information criterion (AIC), Bayesian information criterion (BIC), visual inspection and clinical plausibility of the extrapolations. Finally, the best fitting distribution parameters for the PFS and OS data in both two arms were Log-logistic distributions (survival function: $S\left(t\right)=\frac{1}{\left[1+{\left(\lambda t\right)}^{\gamma }\right]}$) (see Table 1 and Supplementary Tables S1, S2). The exploration and fitting of PFS and OS were displayed in Figure 2 and Supplementary Figures S1, S2. To assess the impact brought by difference survival models, we chose Weibull models (survival function:*S*(*t*) = *EXP*(−λ*t*^γ^)) in scenario analysis (Supplementary Table S3). Taken PFS K-M curves for example, on basis of parametric distributions values (λPFS and γPFS), we could calculate the time-dependency transition probability from PFS state to the next PFS state (pFTF) by using formula: $pFTF=\frac{S\left(t\right)}{S\left(t-1\right)}$. We assumed that the age-specific background mortality derived from China population (9) was equal to the probability of transition from the PFS state to death state (pFTD), so the transition probability from PFS state to PD state (pFTP) was 1- pFTF- pFTD. Similar method described by previous study (10) was used to calculate the other two transition probability.

**Table 1:** Best fit and the values of the parameters.

	**Best fitting**	**Scale (λ)**	**Shape (γ)**
**ITT population**
SC, OS	Log-logistic	0.0274	1.336
SC, PFS	Log-logistic	0.0747	1.662
PC, OS	Log-logistic	0.0425	1.702
PC, PFS	Log-logistic	0.1321	2.142
**Subgroup**
NSQ, SC, PFS	Log-logistic	0.0741	1.604
NSQ, PC, PFS	Log-logistic	0.1147	2.000
SQ, SC, PFS	Log-logistic	0.0769	1.760
SQ, PC, PFS	Log-logistic	0.1586	2.636
PD-L1 <1%, SC, PFS	Log-logistic	0.0893	1.822
PD-L1 <1%, PC, PFS	Log-logistic	0.1339	2.650
PD-L1≥1%, SC, PFS	Log-logistic	0.0641	1.474
PD-L1≥1%, PC, PFS	Log-logistic	0.1324	1.889

ITT, intention-to-treat; SC, sugemalimab plus chemotherapy; PC, placebo plus chemotherapy; PFS, progression-free survival; OS, overall survival; SQ, squamous; NSQ, non- squamous; PD-L1, programmed death ligand 1.

### Cost and utility

Only direct medical costs, calculated from the perspective of the Chinese healthcare system, including costs of drug (first-line and subsequent treatment), and management of serious adverse events (SAEs, grade 3–4), routine follow-up, terminal care in end of life and best supportive care (BSC). As the proportion of male in original trail was 79% in SC arm and 81% in PC arm. To simplify calculating dosage amounts, we assumed a 63-year-old male patient with an average weight of 69.6 Kg, height of 1.69 m, and 70 mL/min creatinine clearance (11, 12), resulting in a body surface area (BSA) of 1.80 m^2^. Currently, sugemalimab patient assistance program (PAP) was conducted for eligible patients to improve the drug affordability in China (13). The PAP supports patients to pay for 2 cycles of sugemalimab, followed by 2 cycles of free sugemalimab (2+2); and then pay for 2 cycles, followed by 11 cycles of donations (2+11); and then pay for 2 cycles, followed by 8 cycles of donations (2+8), which will be considered in scenario analysis. Once disease progressed, patients could receive subsequent therapy and supportive care. As described in GEMSTONE-302 trial, 44.1 and 62.3% of all patients received at least one subsequent treatment in SC and PC arm, respectively. For simplification, we assumed that patients who progressed would receive docetaxel (75 mg/m^2^, Q3W) or tislelizumab (200 mg, Q3W) or suge (1,200 mg, Q3W) or BSC. The detailed proportion of patients receiving subsequent treatment were shown in Supplementary Table S4. In addition, a 2-year maximum treatment duration of sugemalimab and tislelizumab was taken into consideration based on previous studies (14). Only grade 3 or 4 SAEs with an incidence of >5% were considered, including anemia, leukopenia, neutropenia, and thrombocytopenia. The costs related to SAEs were calculated by multiplying the incidence of the SAEs by the costs of managing the SAEs per event. All costs were derived from local hospitals, median price of the winning bid product derived from the Chinese Drug Bidding Database by the YaoZH (www.yaozh.com) (15) or previously published literature.

Health utility values for each health state in our model were derived from previously published studies (16), as no health-related quality of life (HRQoL) was published in clinical trial. We set the health utility values estimates for PFS and PD was 0.75 and 0.59, respectively (16). The disutility values of the following SAEs of grade 3 or 4 were considered: anemia, leukopenia, neutropenia, and thrombocytopenia. Disutility was calculated by multiplying SAEs incidences sourced from GEMSTONE-302 trial and their associated disutility values. Furthermore, a half-cycle correction was implemented to the outcomes, according to the TreeAge Pro 2011 manual and China Guidelines for Pharmacoeconomic Evaluation (7).

### Scenario and sensitivity analysis

Scenario, one-way deterministic and probabilistic sensitivity analysis (PSA) were performed to assess the robustness of model outcomes. In scenario analysis, different time horizons (3–10 years), cost of sugemalimab, median duration of first-line treatment and choice of Weibull survival models were considered. In one-way sensitivity analysis, relevant parameters that had substantial impact on ICER, were tested orderly at the upper and lower limits of plausible ranges (±20% of the base-case value or the lowest and highest bid price), which were listed and illustrated in Table 2. The results of one-way sensitivity analysis were graphed in the tornado diagram. PSA was performed to determine the effects of uncertainty in all model parameters simultaneously *via* 1000 Monte Carlo simulations, which were illustrated in cost-effectiveness acceptability curves. The parametric distribution assumptions in PSA were based on the recommended guidelines in Decision Modeling for Health Economic Evaluation (19). The key parameters input our model were shown in Tables 1, 2.

**Table 2:** Model inputs: base case values, ranges, and distributions for sensitivity analysis.

**Parameters**	**Base case**	**Range**	**Distribution**	**Source**
**Costs (US $)**
Sugemalimab (600 mg)	1,918.6	Fixed	Fixed in PSA	(15)
Carboplatin (100 mg)	8.0	4.1–30.4	Gamma	(15)
Paclitaxel (100 mg)	35.1	19.6–83.2	Gamma	(15)
Pemetrexed (100 mg)	123.7	34.7–332.1	Gamma	(15)
Docetaxel (100 mg)	50.4	38.9–705.4	Gamma	(15)
Tislelizumab (100 mg)	224.8	179.8–269.8	Gamma	(15)
**Costs of SAEs (per event)**
Anemia	537	478–585	Gamma	(17)
Leukopenia	466	415–508	Gamma	(17)
Neutropenia	466	415–508	Gamma	(17)
Thrombocytopenia	6,397	5,117–7,676	Gamma	(17)
Routine follow-up cost per cycle	55.6	27.8–83.4	Gamma	(18)
BSC cost per cycle	337.5	168.7–506.2	Gamma	(18)
Terminal care per cycle	2,627.8	1,313.9–3,941.7	Gamma	(18)
**Risks of SAEs in SC arm (grade 3 or 4) %**
Anemia	13	10.4–15.6	Beta	(4)
Leukopenia	14	11.2–16.8	Beta	(4)
Neutropenia	33	26.4–39.6	Beta	(4)
Thrombocytopenia	11	8.8–13.2	Beta	(4)
**Risks of SAEs in PC arm (grade 3 or 4) %**
Anemia	12	9.6-14.4	Beta	(4)
Leukopenia	17	13.6-20.4	Beta	(4)
Neutropenia	33	26.4-39.6	Beta	(4)
Thrombocytopenia	10	8.0-12.0	Beta	(4)
**Utility values**
Utility of PFS	0.75	0.71–0.85	Beta	(16)
Utility of PD	0.59	0.47–0.71	Beta	(16)
**Disutility of SAEs**
Anemia	0.07	0.06–0.09	Beta	(16)
Leukopenia	0.2	0.16–0.24	Beta	(16)
Neutropenia	0.2	0.16–0.24	Beta	(16)
Thrombocytopenia	0.11	0.09–0.13	Beta	(16)
Discount rate (%)	5	0–8	Fixed in PSA	(7)
**Others**
Subsequent tislelizumab in SC arm (%)	9.1	7.3–10.9	Beta	(4)
Subsequent sugemalimab in SC arm (%)	5.6	4.5–6.7	Beta	(4)
Subsequent docetaxel in SC arm (%)	29.4	23.5–35.3	Beta	estimated
BSC in SC arm	55.9	44.7–67.1	Beta	estimated
Subsequent tislelizumab in PC arm (%)	12.6	10.1–15.1	Beta	(4)
Subsequent sugemalimab in PC arm (%)	27.7	22.2–33.2	Beta	(4)
Subsequent docetaxel in PC arm (%)	22.0	17.6–26.4	Beta	estimated
BSC in PC arm	37.7	30.2–45.2	Beta	estimated

SAEs, serious adverse events; BSC, best supportive care; SC, sugemalimab plus chemotherapy; PC, placebo plus chemotherapy; PFS, progression-free survival; PD, progressed disease; PSA, probabilistic sensitivity analysis.

### Subgroup analysis

A subgroup analysis was performed for the four population groups (NSQ, SQ, PD-L1<1%, PD-L1≥1%) included in the clinical trial to estimate if SC exhibited better in a particular subgroup in terms of cost effectiveness. PFS curves of each subgroup and total OS curves reported in the trial were included in the model for analysis.

## Results

### Clinical outcomes and base-case analysis

The median PFS (mPFS), OS (mOS), and the 12-, 24-, 30-month survival rate estimated by best fitting 2-parametric models matched the results that observed in GEMSTONE-302 trial (4, 6) satisfactorily. For intention-to-treat (ITT) population, the model estimated mPFS of 9.4 vs. 5.3 months and mOS of 25.5 vs. 16.5 months for the SC arm and the PC arm, respectively. Overall, the mPFS, mOS and the 12-, 24-, 30-month survival rate values of ITT and subgroup population obtained by our model were not significantly different from the clinical trial data (Supplementary Table S5).

The results of model base-case analysis with PAP and without PAP were displayed in Table 3. For ITT population, SC arm compared with PC arm provided an additional $62,404.15 and a gain of 0.57 QALYs, resulting in an ICER of $109,480.97/QALYs gained. Considering the PAP, the ICER declined to $52,327.02/QALYs. At the Chinese cost-effectiveness WTP threshold of $37,663/QALYs, SC was clearly not a cost-effective treatment strategy with or without PAP compared with PC.

**Table 3:** Base-case and subgroup results with PAP and without PAP.

**Population**	**Treatment**	**Cost ($)**	**Incremental cost**	**QALYs**	**Incremental QALYs**	**ICER**
**Without PAP**
ITT	SC	88,338.88	62,404.15	1.59	0.57	109,480.97
	PC	25,934.73	NA	1.02	NA	NA
NSQ	SC	93,587.33	65,460.41	1.61	0.56	116,893.59
	PC	28,126.92	NA	1.05	NA	NA
SQ	SC	80,540.73	55,813.88	1.58	0.58	96,230.83
	PC	24,726.85	NA	1.00	NA	NA
PD-L1 < 1%	SC	82,464.80	55,312.86	1.55	0.53	104,363.89
	PC	27,151.94	NA	1.02	NA	NA
PD-L1≥1%	SC	92,502.14	67,070.65	1.64	0.61	109,951.89
	PC	25,431.49	NA	1.03	NA	NA
**With PAP**
ITT	SC	47,144.45	29,826.40	1.59	0.57	52,327.02
	PC	17,318.05	NA	1.02	NA	NA
NSQ	SC	52,113.73	31,589.96	1.61	0.56	56,410.64
	PC	20,523.77	NA	1.05	NA	NA
SQ	SC	40,104.43	25,807.08	1.58	0.58	44,494.97
	PC	14,297.35	NA	1.00	NA	NA
PD-L1 <1%	SC	45,944.47	28,217.76	1.55	0.53	53,241.06
	PC	17,726.71	NA	1.02	NA	
PD-L1≥1%	SC	47,753.76	30,647.88	1.64	0.61	50,242.43
	PC	17,105.88	NA	1.03	NA	NA

ITT, intention-to-treat; QALYs, quality-adjusted life years; ICER, incremental cost-effectiveness ratio; SC, sugemalimab plus chemotherapy; PC, placebo plus chemotherapy; SQ, squamous; NSQ, non- squamous; PD-L1, programmed death ligand 1; NA, not applicable.

### Subgroup analysis

Compared with the PC arm, SC arm resulted in ICER values of $116,893.59, $96,230.83, $104,363.89, and $ 109,951.89 for the population with a tumor pathological type NSQ, SQ and a PD-L1 expression level<1, ≥1, respectively (Table 3). When considering the PAP, the ICER values decreased significantly, but did not change the economic evaluation results (Table 3). It is clear that the SC is not cost-effective in first-line setting for advanced NSCLC patients regardless of PD-L1 expression level and tumor pathological type.

### Sensitivity analysis

**Figure 3:**
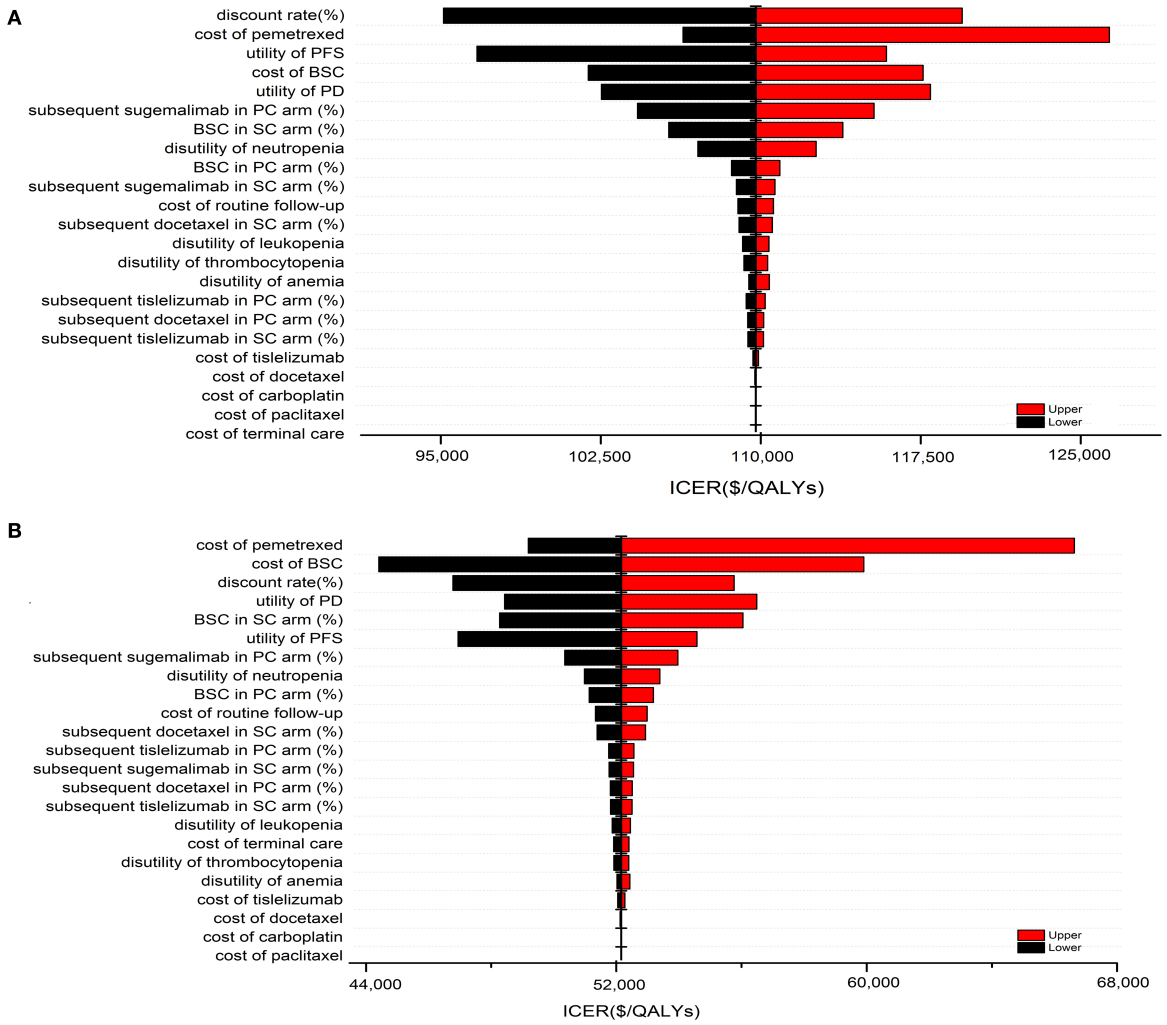
One-way sensitivity analysis of SC in comparison with PC for the ITT population without PAP **(A)** and with PAP **(B)**.

**Figure 4:**
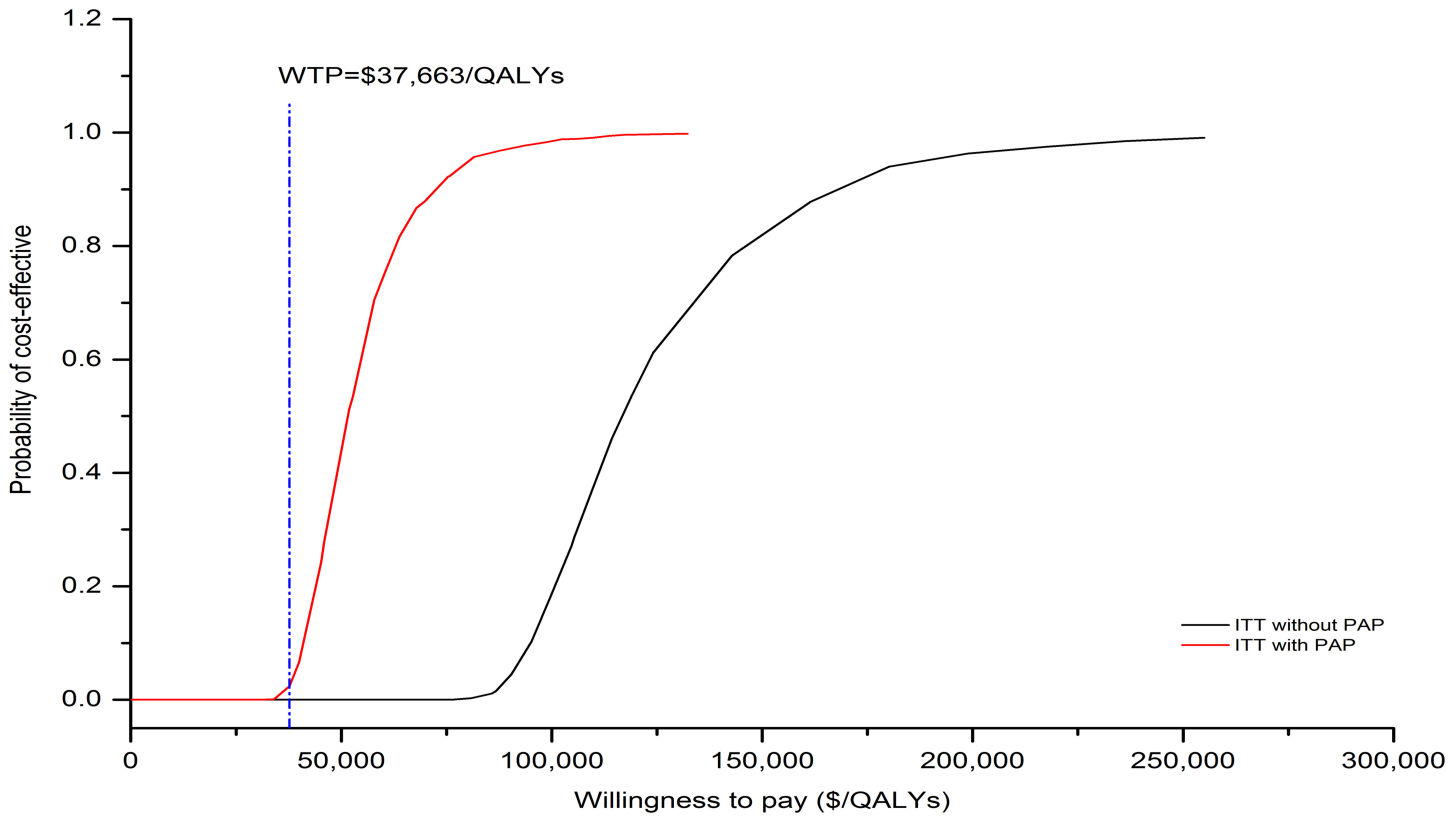
The cost-effectiveness acceptability curves for SC strategy compared to the PC strategy without PAP and with PAP.

The results of one-way sensitivity analysis were shown in a tornado diagram revealing the impact of each key parameter extreme variations on ICER (Figure 3 and Supplementary Figures S3, S4). Parameters with the greatest impact on the ICER were similar among the ITT and the subgroup populations: the discount rate, unit cost of pemetrexed and BSC, the utility of PFS and PD, the proportion of subsequent sugemalimab in PC arm and subsequent BSC in SC arm (Figure 3 and Supplementary Figures S3, S4). However, none of the tested variables' upper or lower limits were able to change the cost-effective treatment strategy from PC to SC, with the ICER above the thresholds. The results of PSA indicating that the probability of the SC arm being cost-effective compared with the PC arm was 0% at a WTP of $37,663/QALYs for the ITT population with or without PAP (Figure 4). The PSA results of subgroup were consistent with those of the ITT population. The results of scenario analysis indicated that the change of parameters, such as the time horizons, cost of sugemalimab, median duration of first-line treatment and choice of Weibull survival models did not affect the stability of our model (Table 4).

**Table 4:** Results of scenario analysis.

**Scenario**	**Incremental cost ($)**	**Incremental QALYs**	**ICER**
**Time horizon**
3 year	55,940.48	0.24	233,085.33
5 year	58,483.90	0.38	153,905.00
8 year	61,016.53	0.51	119,640.25
**Cost of sugemalimab**
80% cost	52,399.55	0.57	91,929.04
60% cost	42,394.95	0.57	74,377.11
40% cost	32,390.34	0.57	56,825.16
**Median duration of first-line treatment**
10 cycles for SC arm and 6 cycles for PC arm	30,464.41	0.57	53,446.33
10 cycles for both arms	29,542.32	0.57	51,828.63
20 cycles for SC arm and 10 cycles for PC arm	49,161.37	0.57	86,248.02
**Choice of Weibull survival models**
ITT	61,276.97	0.47	130,376.53
NSQ	64,669.14	0.45	143,709.20
SQ	54,008.16	0.48	112,517.00
PD-L1 <1%	56,012.05	0.45	124,471.22
PD-L1≥1%	68,598.03	0.50	137,196.06
**Choice of Weibull survival models-with PAP**
ITT	27,866.12	0.47	59,289.62
NSQ	29,602.98	0.45	65,784.40
SQ	23,707.29	0.48	49,390.19
PD-L1 <1%	26,410.07	0.45	58,689.04
PD-L1≥1%	29,997.50	0.50	59,995.00

ITT, intention-to-treat; QALYs, quality-adjusted life years; ICER, incremental cost-effectiveness ratio; SC, sugemalimab plus chemotherapy; PC, placebo plus chemotherapy; SQ, squamous; NSQ, non- squamous; PD-L1, programmed death ligand 1.

## Discussion

As far as we know, this is the first study to estimate the cost-effectiveness of sugemalimab vs. placebo, in combination with platinum-based chemotherapy as first-line treatments of metastatic NSCLC in China. Based on base-case outcomes, the ICER gained by SC arm over a 10-year time frame was not cost-effective compared to PC arm at a WTP threshold of $37,663 in China for ITT and subgroup population. This economic evaluation results were consistent with the previous results of first-line PD-1/PD-L1 antibodies for NSCLC from the perspective of China's healthcare system, including camrelizumab ($63,080/QALYs) (20), pembrolizumab ($92,533/QALYs) (21), atezolizumab ($325,328/QALYs) (22). The results of sensitivity analysis also confirmed the robustness of base-case results.

In GEMSTONE-302 trial, 28% of patients in PC arm crossover to sugemalimab monotherapy after confirmed disease progression, and 13% of patients received other PD-1 antibodies, leading to an effective crossover rate of 41% (4). To ensure the reliability of this cost-effectiveness evaluation results, we included the within-trail crossover of sugemalimab or other PD-1 antibodies between two groups in cost calculation. Meanwhile, to better reflect the cost of first-line treatment in real-world settings, we inputted parameters of the median duration of treatment reported in the GEMSTONE-302 trial in our model for scenario analysis. This scenario analysis results proved the stability of the model. Furthermore, the extrapolation of the survival curve may also affect the stability of the model. So, the choice of a parametric model is crucial. As described by Carroll (23), the Weibull distribution provided better fits to survival data than did other models. A number of cost-effectiveness studies use Weibull distribution to estimate the transition probabilities without considering other distribution models. In our model, the Log-logistic distributions fit the survival curves well, followed by the Weibull, but the significant differences occurred beyond the follow-up time. The Weibull distribution estimated a lower survival rate than Log-logistic distribution. To explore this uncertainty, we compared the effects of those two different parameter distribution models on the results in scenario analysis. The results demonstrated that the selection of Weibull distribution did not change the economic evaluation outcomes. In addition, the assumption of subsequent therapies after disease progression were in line with guideline recommendations and real-word performance, such as the use of tislelizumab or docetaxel (level 1) or BSC for failure of front-line therapy in both arm (24). Overall, this economic evaluation outcomes were based on clinical trial and reasonable clinical assumptions, and the results were credible.

Several limitations should also be noted. First, because of a lack of raw data, we obtain the data from clinical trials, and extrapolation of survival curves beyond the observational time may not accurately reflect the clinical course in the real world. Second, health state utilities used in our model originated from published literature due to the unavailability of quality-of-life data in GEMSTONE-302 trial, which might introduce bias. However, the sensitive analysis suggested that varying the health state utilities did not substantially change the final outcome. Third, the substitution of subgroup OS KM curves for the total OS KM curves, would inevitably introduce bias. In the near future, we will update the study if the updated clinical trial data is available.

## Conclusions

From the perspective of China's healthcare system, the sugemalimab in combination with chemotherapy, regardless of PD-L1 tumor expression level and pathological subtype, was not cost-effective in comparison to chemotherapy as first-line treatment for NSCLC.

## Data availability statement

The original contributions presented in the study are included in the article/Supplementary material, further inquiries can be directed to the corresponding authors.

## Ethics statement

Ethical review and approval was not required for the study of human participants in accordance with the local legislation and institutional requirements. Written informed consent from the patients OR patients legal guardian/next of kin was not required to participate in this study in accordance with the national legislation and the institutional requirements.

## Author contributions

WL design, analyses, and draft the manuscript. LW collect the data and revise the manuscript. All authors reviewed and approved the final version.

## Funding

This work was supported by the Maternal and Child Health Hospital of Hubei Province Research Project (No. 2021SFYM030).

## Conflict of interest

The authors declare that the research was conducted in the absence of any commercial or financial relationships that could be construed as a potential conflict of interest.

## Publisher's note

All claims expressed in this article are solely those of the authors and do not necessarily represent those of their affiliated organizations, or those of the publisher, the editors and the reviewers. Any product that may be evaluated in this article, or claim that may be made by its manufacturer, is not guaranteed or endorsed by the publisher.
