# Supplementary material for: Cost-effectiveness analysis of sugemalimab vs. placebo, in combination with chemotherapy, for treatment of first-line metastatic NSCLC in China
Source: Front Public Health. 2022 Nov 3;10:1015702. doi: 10.3389/fpubh.2022.1015702 (PMC9670176; doi:10.3389/fpubh.2022.1015702)
Supplement: Supplementary file 1 [file Table_1.DOCX]

| **Supplementary Table S1: AIC and BIC of 5 parametric distributions for OS and PFS curves** | | | | | |
| --- | --- | --- | --- | --- | --- |
|  | SC | |  | PC | |
|  | AIC | BIC |  | AIC | BIC |
| **OS** | | | | | |
| Exponential | 1526.52 | 1530.29 |  | 869.09 | 872.16 |
| Weibull | 1525.61 | 1533.14 |  | 861.16 | 867.29 |
| Gompertz | 1527.88 | 1535.41 |  | 867.55 | 873.69 |
| Log-logistic | 1523.65^*^ | 1530.19^*^ |  | 856.48^*^ | 862.62^*^ |
| Log-normal | 1526.96 | 1534.50 |  | 853.69 | 859.83 |
| **PFS** | | | | | |
| Exponential | 1741.30 | 1745.07 |  | 882.69 | 885.76 |
| Weibull | 1729.76 | 1737.30 |  | 867.93 | 874.07 |
| Gompertz | 1740.90 | 1748.43 |  | 882.58 | 888.72 |
| Log-logistic | 1717.28^*^ | 1724.82^*^ |  | 847.10^*^ | 853.23^*^ |
| Log-normal | 1720.07 | 1727.61 |  | 851.67 | 857.81 |
| *, best fitted model; SC: sugemalimab plus chemotherapy; PC: placebo plus chemotherapy; AIC, Akaike information criterion; BIC, Bayesian information criterion; PFS, progression-free survival; OS, overall survival. | | | | | |
