# Supplementary material for: Cost-effectiveness analysis of sugemalimab vs. placebo, in combination with chemotherapy, for treatment of first-line metastatic NSCLC in China
Source: Front Public Health. 2022 Nov 3;10:1015702. doi: 10.3389/fpubh.2022.1015702 (PMC9670176; doi:10.3389/fpubh.2022.1015702)
Supplement: Supplementary file 2 [file Table_2.docx]

| **Supplementary Table S2: AIC and BIC of 5 parametric distributions for subgroup PFS curves.** | | | | | |
| --- | --- | --- | --- | --- | --- |
|  | SC | |  | PC | |
| subgroup | AIC | BIC |  | AIC | BIC |
| **NSQ** | | | | | |
| Exponential | 1,013.55 | 1016.80 |  | 538.27 | 540.84 |
| Weibull | 1009.68 | 1016.18 |  | 532.26 | 537.39 |
| Gompertz | 1015.18 | 1021.69 |  | 539.29 | 544.42 |
| Log-logistic | 1000.78^*^ | 1007.28^*^ |  | 521.84^*^ | 526.97^*^ |
| Log-normal | 997.61 | 1004.12 |  | 522.77 | 527.90 |
| **SQ** | | | | | |
| Exponential | 726.59 | 729.45 |  | 334.97 | 337.12 |
| Weibull | 720.32 | 726.04 |  | 320.85 | 325.13 |
| Gompertz | 725.86 | 731.58 |  | 332.48 | 336.77 |
| Log-logistic | 716.24^*^ | 721.96^*^ |  | 312.26^*^ | 316.54^*^ |
| Log-normal | 721.65 | 727.37 |  | 315.29 | 319.57 |
| **PD-L1<1%** | | | | | |
| Exponential | 803.38 | 806.20 |  | 377.62 | 379.77 |
| Weibull | 801.24 | 806.88 |  | 366.16 | 370.48 |
| Gompertz | 805.35 | 810.99 |  | 376.91 | 381.23 |
| Log-logistic | 786.50^*^ | 792.14^*^ |  | 351.63^*^ | 355.95^*^ |
| Log-normal | 790.43 | 796.07 |  | 351.42 | 355.73 |
| **PD-L1≥1%** | | | | | |
| Exponential | 1187.39 | 1190.67 |  | 576.78 | 579.34 |
| Weibull | 1188.37 | 1194.92 |  | 577.56 | 582.67 |
| Gompertz | 1188.48 | 1195.04 |  | 576.53 | 581.64 |
| Log-logistic | 1175.43^*^ | 1181.98^*^ |  | 556.63^*^ | 561.74^*^ |
| Log-normal | 1172.28 | 1178.84 |  | 559.87 | 564.98 |
| *, best fitted model; SC: sugemalimab plus chemotherapy; PC: placebo plus chemotherapy; AIC, Akaike information criterion; BIC, Bayesian information criterion; PFS, progression-free survival; OS, overall survival. | | | | | |
