# Supplementary material for: Cost-effectiveness analysis of sugemalimab vs. placebo, in combination with chemotherapy, for treatment of first-line metastatic NSCLC in China
Source: Front Public Health. 2022 Nov 3;10:1015702. doi: 10.3389/fpubh.2022.1015702 (PMC9670176; doi:10.3389/fpubh.2022.1015702)
Supplement: Supplementary file 3 [file Table_3.DOCX]

| **Table S3:** The parameter values of Weibull models in scenario analysis. | | | |
| --- | --- | --- | --- |
|  | Model | Scale (λ) | Shape (γ) |
| **ITT population** | | | |
| SC, OS | Weibull | 0.0117 | 1.1361 |
| SC, PFS | Weibull | 0.0249 | 1.2464 |
| PC, OS | Weibull | 0.0093 | 1.3383 |
| PC, PFS | Weibull | 0.0399 | 1.3427 |
| **subgroup** | | | |
| NSQ, SC, PFS | Weibull | 0.0271 | 1.2075 |
| NSQ, PC, PFS | Weibull | 0.0351 | 1.3126 |
| SQ, SC, PFS | Weibull | 0.0221 | 1.3092 |
| SQ, PC, PFS | Weibull | 0.0344 | 1.5772 |
| PD-L1<1%, SC, PFS | Weibull | 0.0353 | 1.1774 |
| PD-L1<1%, PC, PFS | Weibull | 0.0300 | 1.4743 |
| PD-L1≥1%, SC, PFS | Weibull | 0.0320 | 1.0752 |
| PD-L1≥1%, PC, PFS | Weibull | 0.0652 | 1.0985 |
| SC, sugemalimab plus chemotherapy; PC, placebo plus chemotherapy; PFS, progression-free survival; OS, overall survival; SQ, squamous; NSQ, non- squamous; PD-L1, programmed death ligand 1 | | | |
