# Supplementary material for: Cost-effectiveness analysis of sugemalimab vs. placebo, in combination with chemotherapy, for treatment of first-line metastatic NSCLC in China
Source: Front Public Health. 2022 Nov 3;10:1015702. doi: 10.3389/fpubh.2022.1015702 (PMC9670176; doi:10.3389/fpubh.2022.1015702)
Supplement: Supplementary file 4 [file Table_4.DOCX]

| **Supplementary Table S4: proportion of patients receiving subsequent therapy.** | | |
| --- | --- | --- |
| Treatment | SC arm (%) | PC arm (%) |
| tislelizumab | 9.1 | 12.6 |
| sugemalimab | 5.6 | 27.7 |
| docetaxel | 29.4 | 22.0 |
| BSC | 55.9 | 37.7 |
| SC, sugemalimab plus chemotherapy; PC, placebo plus chemotherapy; BSC, best supportive care | | |
