# Supplementary material for: Cost-effectiveness analysis of sugemalimab vs. placebo, in combination with chemotherapy, for treatment of first-line metastatic NSCLC in China
Source: Front Public Health. 2022 Nov 3;10:1015702. doi: 10.3389/fpubh.2022.1015702 (PMC9670176; doi:10.3389/fpubh.2022.1015702)
Supplement: Supplementary file 5 [file Table_5.DOCX]

| **Supplementary Table S5: Validation of modeled PFS and OS data using GEMSTONE-302 data.** | | | | | | |
| --- | --- | --- | --- | --- | --- | --- |
| Curve | | | Median  (mo) | 12-mo  (%) | 24-mo (%) | 30-mo (%) |
| **ITT population** | | |  |  |  |  |
| **PFS** | | |  |  |  |  |
| SC arm | | |  |  |  |  |
| Modeled | | | 9.4 | 40.2 | 17.5 | 12.6 |
| GEMSTONE-302 | | | 9.0 | 36.8 | 20.8 | 19.4 |
| PC arm | | |  |  |  |  |
| Modeled | | | 5.3 | 15.0 | 3.9 | 2.4 |
| GEMSTONE-302 | | | 4.9 | 14.8 | 7.3 | NA |
| **OS** | | |  |  |  |  |
| SC arm | | |  |  |  |  |
| Modeled | | | 25.5 | 70.4 | 52.0 | 46.0 |
| GEMSTONE-302 | | | 25.4 | 71.6 | 51.7 | 45.7 |
| PC arm | | |  |  |  |  |
| Modeled | | | 16.5 | 63.5 | 34.9 | 26.4 |
| GEMSTONE-302 | | | 16.9 | 61.4 | 35.6 | 27.7 |
| **NSQ population-PFS** | | |  |  |  |  |
| SC arm | | |  |  |  |  |
| Modeled | | | 9.4 | 40.8 | 18.5 | 13.5 |
| GEMSTONE-302 | | | 9.6 | 36.1 | 22.1 | NA |
| PC arm | | |  |  |  |  |
| Modeled | | | 6.1 | 20.8 | 6.2 | 3.9 |
| GEMSTONE-302 | | | 5.9 | 20.5 | NA | NA |
| **SQ population-PFS** | | |  |  |  |  |
| SC arm | | |  |  |  |  |
| Modeled | | | 9.1 | 38.4 | 15.6 | 10.9 |
| GEMSTONE-302 | | | 8.3 | 36.6 | 14.9 | NA |
| PC arm | | |  |  |  |  |
| Modeled | | | 4.4 | 6.8 | 1.2 | 0.6 |
| GEMSTONE-302 | | | 4.8 | 6.8 | NA | NA |
| **PD-L1<1% population-PFS** | | |  |  |  |  |
| SC arm | | |  |  |  |  |
| Modeled | | | 7.8 | 31.8 | 11.7 | 7.9 |
| GEMSTONE-302 | | | 7.4 | 28.5 | 13.3 | 12.2 |
| PC arm | | |  |  |  |  |
| Modeled | | | 5.2 | 10.2 | 1.8 | 1.0 |
| GEMSTONE-302 | | | 4.9 | 13.6 | 1.7 | NA |
| **PD-L1≥1% population-PFS** | | |  |  |  |  |
| SC arm | | |  |  |  |  |
| Modeled | | | 10.9 | 46.8 | 24.1 | 18.3 |
| GEMSTONE-302 | 10.9 | | | 42.0 | 25.7 | 24.0 |
| PC arm | |  | |  |  |  |
| Modeled | | 5.3 | | 17.8 | 5.5 | 3.6 |
| GEMSTONE-302 | | 4.9 | | 15.5 | 11.1 | NA |
| ITT, intention-to-treat; SC, sugemalimab plus chemotherapy; PC, placebo plus chemotherapy; PFS, progression-free survival; OS, overall survival; SQ, squamous; NSQ, non- squamous; PD-L1, programmed death ligand 1; NA, not applicable | | | | | | |

c
