# Supplementary material for: Cost-effectiveness analysis of sugemalimab vs. placebo, in combination with chemotherapy, for treatment of first-line metastatic NSCLC in China
Source: Front Public Health. 2022 Nov 3;10:1015702. doi: 10.3389/fpubh.2022.1015702 (PMC9670176; doi:10.3389/fpubh.2022.1015702)
Supplement: Supplementary file 6 [file Data_Sheet_1.DOCX]

Supplementary Material

##
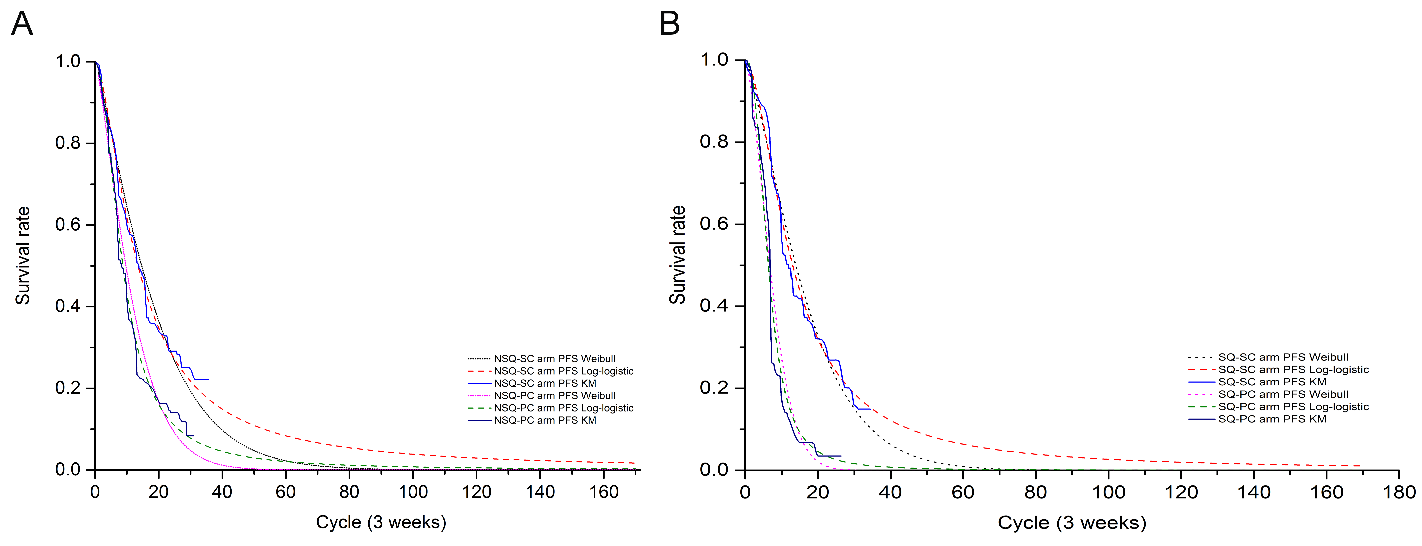
Supplementary Figures

**Supplementary Figure S1:** The exploration and fitting of PFS curves in NSQ subgroup population (A) and in SQ subgroup population (B).


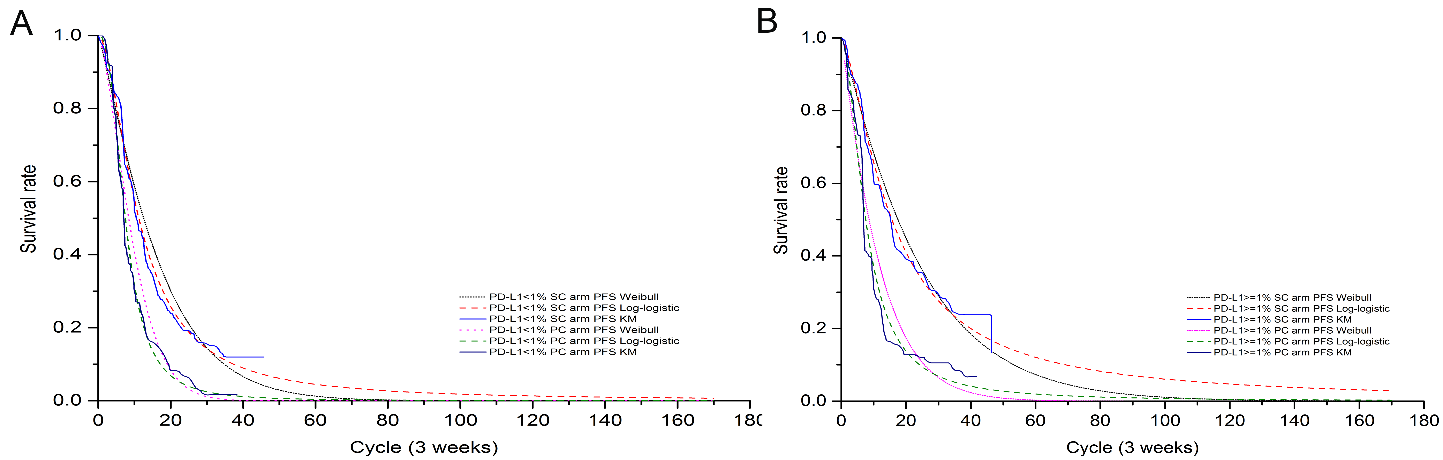


**Supplementary Figure S2:** The exploration and fitting of PFS curves in PD-L1<% subgroup population (A) and in PD-L1>=1% subgroup population (B).


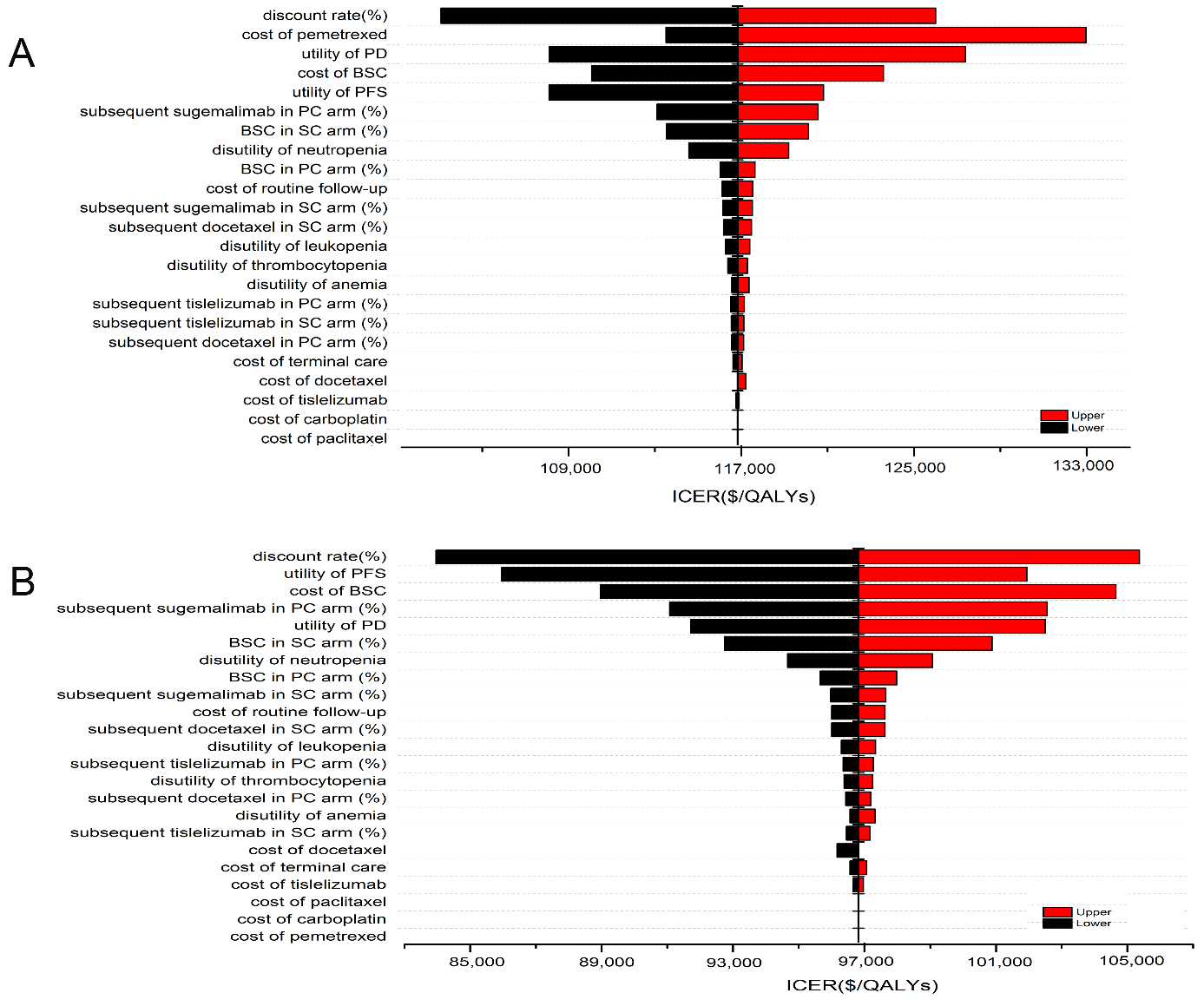


**Supplementary Figure S3:** One-way sensitivity analysis of SC in comparison with PC for the subgroup population without PAP. (A) NSQ population (B) SQ population


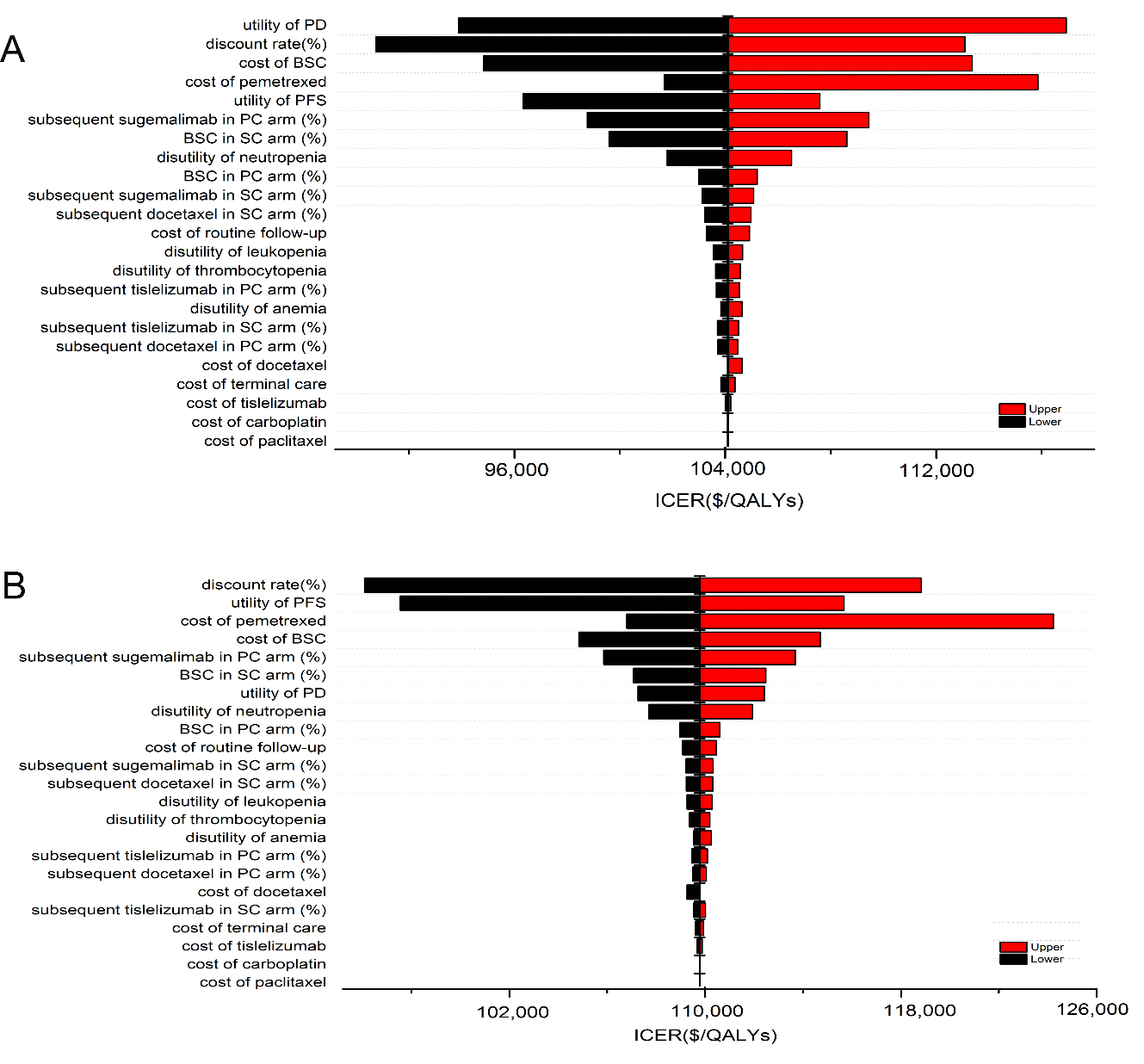


**Supplementary Figure S4:** One-way sensitivity analysis of SC in comparison with PC for the subgroup population without PAP. (A) PD-L1<1% population (B) PD-L1>=1% population
